# Sociodemographic disparities in purchases of fruit drinks with policy relevant front-of-package nutrition claims

**DOI:** 10.1017/S1368980023000691

**Published:** 2023-08

**Authors:** Emily Duffy, Shu Wen Ng, Marissa G Hall, Maxime Bercholz, Natalia Rebolledo, Aviva Musicus, Lindsey Smith Taillie

**Affiliations:** 1 Department of Nutrition, University of North Carolina at Chapel Hill Gillings School of Global Public Health and Carolina Population Center, 123 W Franklin St, Chapel Hill, NC 27516, USA; 2 Department of Health Behavior, University of North Carolina at Chapel Hill Gillings School of Global Public Health and Carolina Population Center, and UNC Lineberger Comprehensive Cancer Center, School of Medicine, Chapel Hill, NC, USA; 3 Carolina Population Center, Chapel Hill, NC, USA; 4 Department of Social and Behavioral Sciences, Harvard T.H. Chan School of Public Health, Boston, MA, USA

**Keywords:** Sugar-sweetened beverage, Nutrition claims, Childhood

## Abstract

**Objective:**

Our objectives were to describe sociodemographic characteristics associated with the purchase of (1) any fruit drinks and (2) fruit drinks with specific front-of-package (FOP) nutrition claims.

**Design:**

Cross-sectional.

**Setting:**

USA

**Participants:**

We merged fruit drink purchasing data from 60 712 household-months from 5233 households with children 0–5 years participating in Nielsen Homescan in 2017 with nutrition claims data. We examined differences in predicted probabilities of purchasing any fruit drinks by race/ethnicity, income and education. We constructed inverse probability (IP) weights based on likelihood of purchasing any fruit drinks. We used IP-weighted multivariable logistic regression models to examine predicted probabilities of purchasing fruit drinks with specific FOP claims.

**Results:**

One-third of households with young children purchased any fruit drinks. Non-Hispanic (NH) Black (51·6 %), Hispanic (36·3 %), lower-income (39·3 %) and lower-educated households (40·9 %) were more likely to purchase any fruit drinks than NH White (31·3 %), higher-income (25·8 %) and higher-educated households (30·3 %) (all *P* < 0·001). In IP-weighted analyses, NH Black households were more likely to purchase fruit drinks with ‘Natural’ and fruit or fruit flavour claims (6·8 % and 3·7 %) than NH White households (4·5 % and 2·7 %) (both *P* < 0·01). Lower- and middle-income (15·0 % and 13·8 %) and lower- and middle-educated households (15·4 % and 14·5 %) were more likely to purchase fruit drinks with ‘100 % Vitamin C’ claims than higher-income (10·8 %) and higher-educated households (12·9 %) (all *P* < 0·025).

**Conclusions:**

We found a higher likelihood of fruit drink purchases in lower-income, lower-educated, NH Black and Hispanic households. Experimental studies should determine if nutrition claims may be contributing to disparities in fruit drink consumption.

Fruit drinks (i.e. fruit-flavoured juice cocktails, cordials, nectars with added caloric sweeteners, not including 100 % juice) are the most common type of sugar-sweetened beverage (SSB) consumed and a top source of added sugar among young children in the USA^(1–3)^. SSB consumption in childhood is not recommended and is associated with a greater risk of diet-related chronic diseases^(4)^. As a result of socioecological factors such as targeted marketing^(5)^, there are disparities in SSB and fruit drink consumption by sociodemographic characteristics such as race/ethnicity and income^(1,6,7)^, such that non-Hispanic (NH) Black and Hispanic as well as children living in households with low incomes have higher intakes of SSB and fruit drinks. Despite the poor nutritional profile of fruit drinks, prior studies have demonstrated that front-of-package (FOP) nutrition claims are essentially universal on fruit drink products purchased by households with young children in the USA, and these claims are not clear indicators of products with an improved nutritional profile and can mislead parents about the healthfulness of fruit drinks^(8–10)^. Parent sociodemographic characteristics are likely associated with propensity to purchase products with certain types of claims, as studies have shown differences in self-reported label and claim use by sociodemographic characteristics^(11,12)^,but this is not well understood.

Parents of young children, in particular, may be reliant on FOP claims to make quick purchasing decisions as they may be distracted or functioning under other constraints while shopping for food^(13,14)^. Additionally, parents state they use claims in making purchases^(15,16)^, and experimental studies with parents have demonstrated that the presence of claims may lead to selection of less healthy foods and beverages and that claims can be equally misleading across categories of parent race/ethnicity, income and education^(17,18)^. While we know there are disparities in fruit drink consumption and in food label usage among different sociodemographic groups, we do not yet know if FOP nutrition claims are an important contributor to observed disparities in fruit drink purchases for children nor do we know which claims are particularly common on products purchased by sociodemographic groups at greatest risk of diet-related chronic diseases.

Understanding the associations between sociodemographic characteristics and purchases of products with specific FOP claims can inform current food labelling regulatory and policy efforts such as the Food Labeling Modernization Act or the US Food and Drug Administration’s (FDA) Nutrition Innovation Strategy. The Nutrition Innovation Strategy states that its overall goal is to prevent death and disease related to poor nutrition^(19)^. If the goals of revised labelling policies and regulations are to have a large health impact and reduce existing health disparities, it is important to understand which claim types may be most common on fruit drinks purchased by groups with the greatest burden of diet-related disease. Given these gaps, the two aims of this study were to examine the association between household sociodemographic characteristics and (1) overall fruit drink purchases and (2) purchases of fruit drinks carrying specific FOP nutrition claims among households with young children (0–5 years) in the USA.

## Methods

### Data source

We used household monthly level food and beverage purchasing data from the Nielsen Homescan panel in 2017. Homescan is a nationally projectable, longitudinal panel dataset of more than 60 000 households across fifty-four metropolitan and twenty-four non-metropolitan US markets. Participating households use a scanner to document their purchases of all foods and beverages with a barcode. Households included must be a permanent residence, report purchases consistently for at least 10 months out of the year and purchase a certain dollar amount of food in a 1-month period ($45 for a single-person household and $135 for households with two or more people). Products purchased are then categorised by a team of nutritionists that are part of our research group into nutritionally relevant food groups and linked to detailed nutrition information using the Nutrition Facts Label. This process has been described in detail elsewhere^(20)^. A small percentage (about 5 %) of food purchases, including about 3 % of fruit drink purchases, could not be matched directly to nutrition information and are excluded from this study.

### Sample

**Fig. 1:**
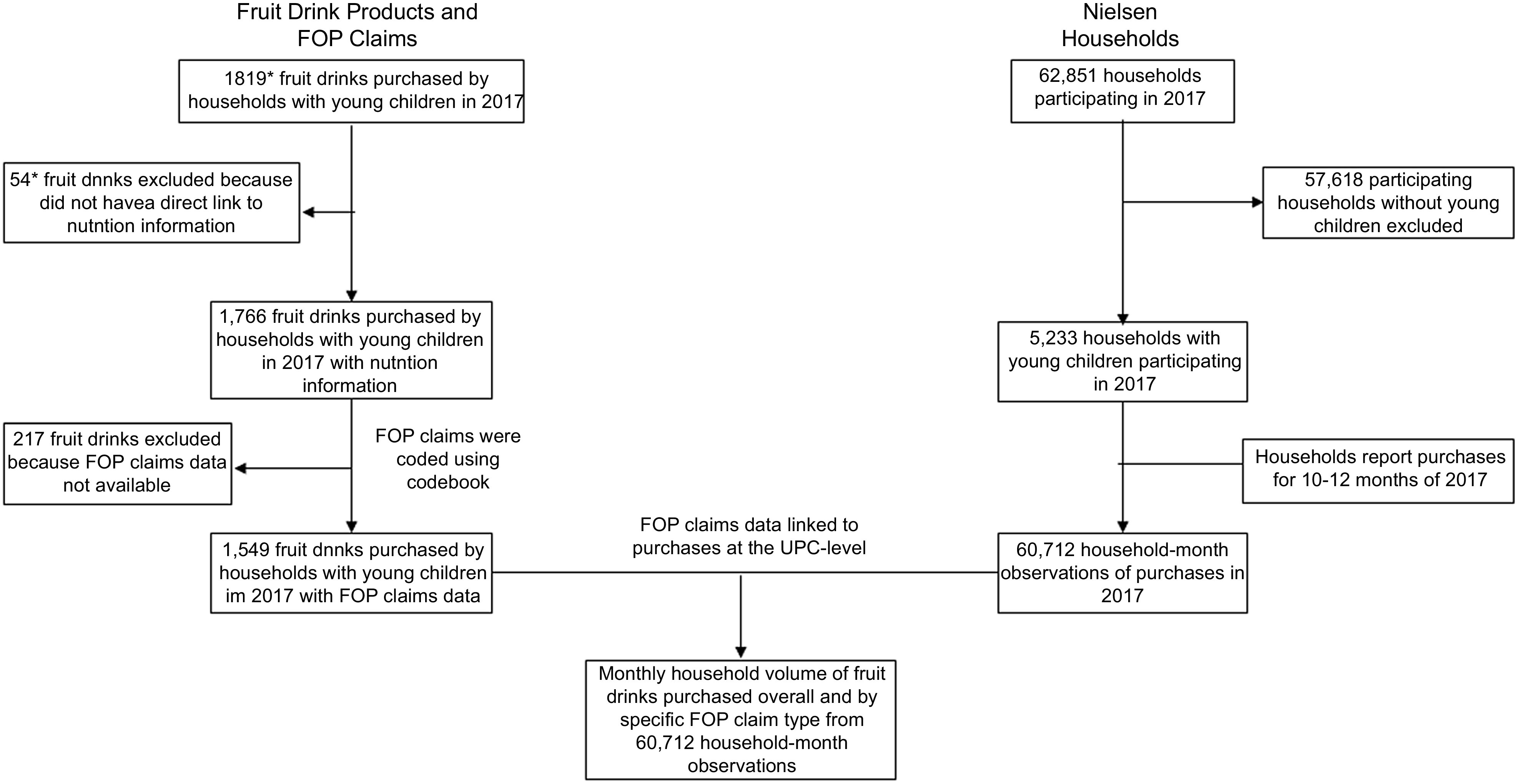
Flow chart describing selection of fruit drinks and households in sample *These values are estimates based on ~3 % of all fruit drinks purchased in 2017 not having a direct link to nutrition information. FOP, front-of-package; UPC, universal product code.

In 2017, 62 851 households participated in Homescan. Given this study’s focus on young children, only households with children 0–5 years old were included (*n* 5233). This study uses household-month observations instead of annual observations because fruit drinks are commonly consumed and purchased by households with young children, so over the course of 1 year most households will have purchased > 0 ml of fruit drinks. Because we are using a binary outcome for most of our models (i.e. purchased yes or no), using monthly observations allows us to better differentiate between purchasing and non-purchasing households. Among households with young children, there were 60 712 household-month observations in 2017 (Fig. 1). We only included household purchases of fruit drinks in our analysis.

### Fruit drink product sample and linking purchases to claims data

Product-level FOP nutrition claims data were previously collected for fruit drinks purchased by Homescan households with young children in 2017 with a direct match at the product level to nutrition information (*n* 1766 fruit drink products)^(8)^. Of these 1766 products, 217 products did not have claims data because a legible package label could not be identified to code, the labels were not included in the two label databases (Label Insight and Mintel Global New Products Database) used to code images, or the images of the labels that were coded were posted after 2018, suggesting that the label coded may not be identical to the label on the product purchased by Homescan households in 2017. Only 1·5 % of households purchased fruit drinks with missing FOP claims data, and the mean volume purchased per capita per d of these beverages was 0·32 ml, compared with an overall mean volume purchased per capita per d of 18·0 ml for all fruit drinks, so fruit drinks with missing claims data did not contribute meaningfully to total fruit drink purchases. Therefore, purchases of 1549 fruit drink products were used to determine the predicted probabilities of purchasing and per capita per d volumes of fruit drinks purchased (Fig. 1). Some fruit drinks with claims data were frozen or liquid concentrates and powders. To calculate the as-consumed volumes, reconstitution factors were created and applied. Unique reconstitution factors were created based on preparation methods and product packaging instructions for powders with caloric sweeteners, powders with both caloric sweeteners and non-caloric sweeteners, frozen concentrates, and liquid concentrates.

For the purposes of this study, we focused our analyses on five claim types: ‘100 % Vitamin C’ (present on 20 % of products^(8)^), ‘Natural Flavors’ (on 43 % products), ‘Natural’ (on 7 % of products), ‘No High Fructose Corn Syrup’ (on 7 % of products), and fruit or fruit flavour claims (on 6 % of products) such as ‘Made with Real Oranges’, ‘Made with Whole Fruit’ or ‘Real Flavor from Real Fruit’. The methodology used to determine the presence of these claims is described in detail elsewhere^(8)^. We selected these claims based on prevalence on fruit drinks, policy relevance, potential to mislead consumers and consumer behaviour theory. In terms of prevalence, some claims such as ‘100 % Vitamin C’ and ‘Natural Flavors’ are more common than others such as ‘No High Fructose Corn Syrup’ in the food supply^(8)^. In terms of policy relevance, the FDA is currently considering revisions to the regulations of nutrient content claims such as ‘100 % Vitamin C’ and the definition of ‘Natural’ (which may or may not also include ‘Natural Flavors’^(21)^) through its Nutrition Innovation Strategy and advocates have called for improvements in how these two specific claim types are regulated^(19,22)^. Additionally, claims such as ‘No High Fructose Corn Syrup’, ‘100 % Vitamin C’, and fruit or fruit flavour claims were prioritised based on their impact on fruit drink perceptions and purchase intentions in a pretest of a variety of claims with 1002 adults recruited through Amazon Mechanical Turk^(17)^. Relative to a control fruit drink with no claim, fruit drinks with these claims were more likely to lead participants to select a fruit drink over a 100 % fruit juice than the other five claim types tested and thus warrant deeper examination using real-world purchasing data given the influence they appear to have on consumers. Finally, based on consumer behaviour theory that consumers respond differently to presence-framed claims (e.g. ‘100 % Vitamin C’) and absence-framed claims (e.g. ‘No High Fructose Corn Syrup’) based on consumer characteristics or the food product carrying the claim, we selected both types for inclusion^(11,23)^. The product-level FOP claims data for these five claims were linked to the monthly food purchasing data, producing the primary outcome, household volume of fruit drinks with each of the five claim types purchased (Fig. 1).

### Outcomes

Our outcomes included total per capita per d volume of any fruit drinks purchased as well as total per capita per d volume of fruit drinks with each of the five specific claim types purchased. To derive per capita outcomes, each household’s monthly volume of fruit drinks purchased was divided by their household size and the number of days in the month. Total per purchaser per d volumes of fruit drinks for a given month were calculated by limiting the sample to only households that purchased any fruit drinks and doing the same per capita per d calculations.

### Covariates

The sociodemographic characteristics of primary interest were head of household race/ethnicity, female head of household education and household income. Other covariates used include female head of household age, number of children (0–18 years) in the household and number of adults in the household. Female head of household’s age and education were used as females are most often the primary food shopper^(24)^. All sociodemographic characteristics were measured in Homescan’s annual demographic survey.

Female head of household education was measured as grade school, some high school, graduated high school, some college, graduated college, post-college graduate or no female head of household/unknown. We recategorised these values as high school education or less (lower), some college (middle), and college education or more (higher), because prior studies examining food label use and education have found the largest differences in these three groups^(11,12,25)^.

Race was measured using racial self-classification^(26)^ based on four closed-ended options: White, Black, Asian and other race. Hispanic ethnicity was measured as a dichotomous variable. We recategorised race and Hispanic ethnicity into five groups: NH White, NH Black, NH Asian, NH other race and Hispanic. Race and Hispanic ethnicity in our analyses are not indicators of biological differences but are representations of the sociopolitical processes that differentially impact individuals^(27)^. We do not know which other race categories are included in the other race category; however, it is important to note that this group is very heterogeneous and does not necessarily represent a set of shared characteristics or experiences. Female household head age was modelled categorically based on quartiles due to functional form assessment. If there was no female head of household (1·7 % households), male head of household information was used. We used reported household size to recalculate continuous household income as a proportion of the Federal Poverty Level (FPL) index and created three categories of FPL index: less than 185 %, 185–400 % and greater than 400 % FPL. We selected these categories based on eligibility for federal nutrition assistance and other income support programmes as well as assessment of functional form which involved comparing the log-likelihood and AIC of models in which FPL index was categorised as either a continuous or categorical variable. We also used indicator variables for month in all adjusted models to account for potential seasonality of purchasing behaviours.

### Analysis

We conducted all analyses on Stata 16^(28)^. For our first aim of examining sociodemographic characteristics associated with fruit drink purchases, regardless of claim type, we used unadjusted logistic regression to compare the proportion of households purchasing any fruit drinks (> 0 ml per capita per d) by household sociodemographic characteristics. We used Stata’s margins command to obtain the predicted probabilities of being a fruit drink purchaser. We also compared the mean per purchaser per d volume (i.e. the mean per capita per d among only fruit drink purchasing households) of fruit drinks purchased by household sociodemographic characteristics using unadjusted generalised linear models with a γ distribution and a log link. For our second aim of examining sociodemographic characteristics associated with fruit drinks with specific FOP claims, we first used multivariable-adjusted logistic regression and the margins command to examine differences in the predicted probability of purchasing fruit drinks with each of the five FOP claims of interest (> 0 ml per capita per d) by household race/ethnicity, income and education accounting for other household-level confounders. These models were adjusted for head of household race/ethnicity, female head of household education, female head of household age, household FPL index, number of children, and number of adults, and month.

Households with certain sociodemographic characteristics were more likely than others to purchase any fruit drinks, potentially introducing selection bias into our analyses of household characteristics associated with purchasing fruit drinks with a specific FOP claims^(29)^. To account for this selectivity of being a fruit drink purchaser, we created inverse probability (IP) weights based on each household’s likelihood of purchasing any fruit drinks (online Supplementary S1 File, S1 Table, S2 Table)^(30,31)^. We then used IP-weighted multivariable-adjusted logistic regression models to examine differences in the predicted probabilities of purchasing fruit drinks with each of the five FOP claims, making all households equally likely to purchase any fruit drinks (conditional on the observed household characteristics) in the weighted sample and potentially reducing this selection bias. Weighting allowed us to utilise all households’ data and prevent further bias introduced by excluding non-purchasing households from analyses^(30)^. Weighting also allowed us to make inferences about the population of all households regardless of their individual propensities to purchase fruit drinks. The IP-weighted models were adjusted for head of household race/ethnicity, female head of household education, female head of household age, household FPL index, number of children, and number of adults, and month.

All standard errors were adjusted for clustering of household-month observations at the household level. The level of significance for all statistical tests was determined by the Holm method for adjusting for multiple comparisons^(32)^. This method controls the familywise type 1 error rate. We applied this method to control the familywise type 1 error rate at a level of 0·05 within each outcome and household characteristic (e.g. outcome: probability of purchasing a fruit drink with a ‘Natural’ claim, and household characteristic: head of household race/ethnicity). This study was reviewed by UNC Chapel Hill’s Institutional Review Board and deemed non-human subjects research, so a consent process was not required.

## Results

### Sociodemographic characteristics of the sample

In this sample of 60 712 household-month observations from 5233 unique households with young children, the majority of households had a NH White head of household, had an income between 185 % and 400 % of the FPL index, and had a female head of household with a college education or more (Table 1). The mean age of female heads of household was 37·5 (sd: 8·1) years. Households had a mean of 2·1 (sd: 1·1) children.

**Table 1:** Sample sociodemographic characteristics, 2017 (*n* 60 712 household-month observations from 5233 unique households with young children)

	%
Head of household race/ethnicity	
NH White	70·3
NH Black	9·4
NH Asian	5·2
NH other race	2·8
Hispanic	12·2
Female head of household education	
College degree or more	60·3
Some college	26·5
High school degree or less	13·3
Household income	
Greater than 400 % FPL	15·1
185–400 % FPL	55·9
Less than 185 % FPL	29·1
Female head of household age	
Less than 34 years	31·5
34–36 years	22·4
37–40 years	22·1
Greater than 40 years	23·9
Mean	37·5
sd	8·1
Number of children (0–18 years) in household	
One	31·7
Two	39·3
Three	18·7
Four or more	10·4
Mean	2·1
sd	1·1

NH, non-Hispanic; FPL, Federal Poverty Level.

Nielsen disclaimer: Authors’ calculations based in part on data reported by Nielsen through its Homescan Services for all food categories, including beverages and alcohol for the 2017 period across the US market. The Nielsen Company, 2017. Nielsen is not responsible for and had no role in preparing the results reported herein.

### Purchases of any fruit drinks

On average, 33·5 % of households purchased any fruit drinks in a given month, regardless of claim type, and among purchasing households the mean volume purchased was 53·8 ml per person per d (about 1/4 cup). NH Black households (51·6 %) and Hispanic households (36·3 %) were more likely to purchase fruit drinks than NH White households (31·3 %) (both *P* < 0·001). NH Asian households (23·1 %, *P* < 0·001) were less likely to purchase fruit drinks compared with NH White households (Table 2). Among purchasing households, NH Black households on average purchased more fruit drinks per purchaser per d (63·6 ml) than NH White households (51·7 ml, *P* < 0·001) (Table 2). Middle- (32·5 %) and lower-income (39·3 %) households were more likely to purchase fruit drinks than higher-income households (25·8 %) (both *P* < 0·001). Lower-income households purchased more fruit drinks per purchaser per d (58·4 ml) than higher-income households (49·5 ml, *P* = 0·001). Middle- (37·1 %) and lower-educated households (40·9 %) were more likely to purchase fruit drinks than higher-educated households (30·3 %) (both *P* < 0·001). Lower-educated households purchased more fruit drinks per purchaser per d (58·6 ml) than higher-educated households (51·5 ml, *P* = 0·006).

**Table 2:** Percent that purchased any fruit drinks and mean volume per purchaser per d by household sociodemographic characteristics (*n* 60 712 household-month observations from 5233 unique households with young children)

Household characteristic	Percent that purchased any fruit drink	95 % CI	*P* value	Mean volume/purchaser/d	95 % CI	*P* value
Overall	33·5 %			53·8		
Head of household race/ethnicity						
NH White (ref)	31·3 %	30·4 %, 32·2 %		51·7	49·6, 53·9	
NH Black	51·6 %	48·9 %, 54·3 %	< 0·001*	63·6	58·9, 68·2	< 0·001*
NH Asian	23·1 %	20·2 %, 26·0 %	< 0·001*	48·1	37·4, 58·8	0·529
NH other race	35·2 %	30·9 %, 39·6 %	0·076	47·2	40·8, 53·6	0·208
Hispanic	36·3 %	34·1 %, 38·5 %	< 0·001*	56·7	52·1, 61·2	0·048
Household income						
Greater than 400 % FPL (ref)	25·8 %	24·0 %, 27·7 %		49·5	45·6, 53·4	
185–400 % FPL	32·5 %	31·5 %, 33·6 %	< 0·001*	51·9	49·6, 54·1	0·311
Less than 185 % FPL	39·3 %	37·8 %, 40·8 %	< 0·001*	58·4	55·1, 61·8	0·001*
Female head of household education						
College degree or more (ref)	30·3 %	29·3 %, 31·2 %		51·5	49·5, 53·5	
Some college	37·1 %	35·6 %, 38·7 %	< 0·001*	55·5	51·8, 59·1	0·057
High school degree or less	40·9 %	38·7 %, 43·1 %	< 0·001*	58·6	53·7, 63·6	0·006*

NH, non-Hispanic; FPL, Federal Poverty Level.

Nielsen disclaimer: Authors’ calculations based in part on data reported by Nielsen through its Homescan Services for all food categories, including beverages and alcohol for the 2017 across the US market. The Nielsen Company, 2017. Nielsen is not responsible for and had no role in preparing the results reported herein.

* Statistically significantly different after using the Holm method to control the familywise error rate at level 0·05 within each outcome and household characteristic. CI not adjusted for multiple comparisons.

### Purchases of fruit drinks with specific front-of-package claims

In unweighted analyses adjusted for household-level characteristics, we found that the differences in purchases of fruit drinks with the five claim types of interest by sociodemographic characteristics (Table 3) mirror those of the probability of purchasing any fruit drinks, highlighting the need to account for the selectivity of being a fruit drink purchaser.

**Table 3:** Adjusted† predicted probabilities and 95 % CI of purchasing > 0 ml of fruit drinks with five specific front-of-package claim types by head of household race/ethnicity, household income or female head of household education (*n* 60 712 household-month observations from 5233 unique households with young children)

Household characteristic	Any fruit drinks	100 % vitamin C claim	95 % CI%	*P* value	Natural claim	95 % CI	*P* value	Natural flavors claim	95 % CI	*P* value	No high fructose corn syrup claim	95 % CI	*P* value	Fruit or fruit flavor claim	95 % CI	*P* value
Overall	33·5 %	14·4 %			5·1 %			19·1 %			4·9 %			3·1 %		
Head of household race/ethnicity																
NH White (ref)	30·8 %	12·7 %	12·1 %, 13·4 %		4·1 %	3·8 %, 4·4 %		17·1 %	16·4 %, 17·8 %		4·4 %	4·1 %, 4·8 %		2·5 %	2·3 %, 2·8 %	
NH Black	51·4 %	22·7 %	20·4 %, 25·0 %	< 0·001*	10·6 %	9·1 %, 12·2 %	< 0·001*	31·4 %	29·0 %, 33·9 %	< 0·001*	6·7 %	5·6 %, 7·9 %	< 0·001*	5·5 %	4·5 %, 6·5 %	< 0·001*
NH Asian	24·7 %	8·3 %	6·5 %, 10·2 %	< 0·001*	4·2 %	2·9 %, 5·5 %	0·867	12·8 %	10·4 %, 15·1 %	0·002*	3·7 %	2·7 %, 4·8 %	0·267	3·2 %	2·1 %, 4·2 %	0·212
NH other race	34·4 %	13·1 %	9·8 %, 16·4 %	0·831	7·3 %	5·0 %, 9·7 %	0·001*	18·6 %	15·0 %, 22·1 %	0·430	3·9 %	2·5 %, 5·3 %	0·497	3·2 %	2·2 %, 4·2 %	0·161
Hispanic	35·5 %	14·3 %	12·7 %, 15·9 %	0·068	5·3 %	4·4 %, 6·2 %	0·008*	19·0 %	17·2 %, 20·8 %	0·051	5·1 %	4·2 %, 5·9 %	0·160	2·8 %	2·2 %, 3·5 %	0·375
Household income																
Greater than 400 % FPL (ref)	28·9 %	9·4 %	8·0 %, 10·8 %		4·7 %	3·8 %, 5·5 %		14·5 %	12·9 %, 16·0 %		4·3 %	3·4 %, 5·2 %		2·1 %	1·6 %, 2·7 %	
185–400 % FPL	32·6 %	13·4 %	12·7 %, 14·2 %	< 0·001*	4·7 %	4·3 %, 5·1 %	0·945	18·1 %	17·3 %, 18·9 %	< 0·001*	4·8 %	4·3 %, 5·2 %	0·368	2·8 %	2·5 %, 3·1 %	0·044
Less than 185 % FPL	35·7 %	16·0 %	14·8 %, 17·1 %	< 0·001*	4·8 %	4·2 %, 5·3 %	0·834	20·7 %	19·5 %, 21·9 %	< 0·001*	4·5 %	4·0 %, 5·1 %	0·657	3·2 %	2·8 %, 3·6 %	0·008*
Female head of household education																
College degree or more (ref)	30·5 %	11·8 %	11·1 %, 12·5 %		4·7 %	4·3 %, 5·1 %		16·6 %	15·8 %, 17·3 %		4·5 %	4·1 %, 4·8 %		2·6 %	2·3 %, 2·9 %	
Some college	35·4 %	15·3 %	14·1 %, 16·5 %	< 0·001*	5·0 %	4·4 %, 5·6 %	0·413	19·9 %	18·7 %, 21·2 %	< 0·001*	4·9 %	4·3 %, 5·5 %	0·241	3·2 %	2·7 %, 3·6 %	0·027
High school degree or less	39·4 %	18·0 %	16·2 %, 19·8 %	< 0·001*	4·5 %	3·7 %, 5·2 %	0·674	23·2 %	21·3 %, 25·1 %	< 0·001*	4·8 %	4·0 %, 5·7 %	0·449	3·1 %	2·5 %, 3·7 %	0·146

NH, non-Hispanic; FPL, Federal Poverty Level.

Nielsen disclaimer: Authors’ calculations based in part on data reported by Nielsen through its Homescan Services for all food categories, including beverages and alcohol for the 2017 periods across the US market. The Nielsen Company, 2017. Nielsen is not responsible for and had no role in preparing the results reported herein.

* Statistically significantly different after using the Holm method to control the familywise error rate at level 0·05 within each outcome and household characteristic. CI not adjusted for multiple comparisons.

† Model was adjusted for head of household race/ethnicity, female head of household education, federal poverty level index, female head of household age, number of adults in the household, number of children in the household and month of observation. se’s are adjusted for clustering at the household level.

In adjusted and IP-weighted analyses accounting for each household’s likelihood of being a fruit drink purchaser, NH Black households were more likely to purchase fruit drinks with ‘Natural’ and fruit or fruit flavour claims (6·8 %, *P* < 0·001 and 3·7 %, *P* = 0·009, respectively) than NH White households (4·5 %, and 2·7 %, respectively) (Table 4). NH other race households were also more likely to purchase fruit drinks with ‘Natural’ claims (7·4 %, *P* = 0·005) than NH White households (4·5 %). There were no significant differences in fruit drinks purchases with specific claims between NH White and Hispanic households. Lower- and middle-income households remained more likely to purchase fruit drinks with ‘100 % Vitamin C’ claims (15·0 %, *P* < 0·001 and 13·8 %, *P* = 0·002, respectively) than higher-income households (10·8 %). Similarly, lower- and middle-educated households were more likely to purchase fruit drinks with ‘100 %Vitamin C’ claims after weighting (15·4 %, *P* = 0·004 and 14·5 %, *P* = 0·018, respectively) than higher-educated households (12·9 %) (Table 4). Lower-educated households were less likely to purchase fruit drinks with ‘Natural’ claims (3·8 %, *P* = 0·002) than higher-educated households (5·2 %). There were no significant differences in the likelihood of purchasing fruit drinks with ‘No High Fructose Corn Syrup’ or ‘Natural Flavor’ claims by household race/ethnicity, income or education after weighting (Table 4).

**Table 4:** Inverse probability-weighted multivariable-adjusted† monthly predicted probabilities, differences in predicted probabilities and 95 % CI of purchasing > 0 ml of fruit drinks with five specific front-of-package claims by head of household race/ethnicity, household income or female head of household education (*n* 60 712 household-month observations from 5233 unique households with young children)

Household characteristic	Any fruit drink	100 % vitamin C	95 % CI	Difference	95 % CI	P value	Natural	95 % CI	Difference	95 % CI	P value	Natural flavour	95 % CI	Difference	95 % CI	P value
Head of household race/ethnicity																
NH White (ref)	30·8 %	13·8 %	13·1 %,14·5 %				4·5 %	4·1 %, 4·9 %				18·6 %	17·9 %, 19·4 %			
NH Black	51·4 %	14·7 %	13·0 %,16·4 %	0·9 %	–0·9 %, 2·8 %	0·309	6·8 %	5·7 %, 8·0 %	2·4 %	1·2 %, 3·5 %	< 0·001*	20·3 %	18·4 %, 22·3 %	1·7 %	–0·4 %, 3·8 %	0·095
NH Asian	24·7 %	11·1 %	8·7 %, 13·4 %	−2·7 %	–5·2 %, –0·2 %	0·048	5·7 %	4·0 %, 7·3 %	1·2 %	–0·5 %, 2·9 %	0·136	17·3 %	14·2 %, 20·3 %	−1·3 %	–4·5 %, 1·8 %	0·420
NH other race	34·4 %	12·5 %	9·3 %, 15·8 %	−1·3 %	–4·5 %, 2·1 %	0·474	7·4 %	4·9 %, 9·9 %	2·9 %	0·4 %, 5·4 %	0·005*	18·0 %	14·6 %, 21·5 %	−0·6 %	–4·1 %, 2·9 %	0·744
Hispanic	35·5 %	13·5 %	11·9 %, 15·0 %	−0·3 %	–2·0 %, 1·4 %	0·714	5·0 %	4·2 %, 5·9 %	0·5 %	–0·4 %, 1·5 %	0·251	17·9 %	16·1 %, 19·6 %	−0·8 %	–2·6 %, 1·1 %	0·437
Household income																
Greater than 400 % FPL (ref)	28·9 %	10·8 %	9·2 %, 12·4 %				5·4 %	4·4 %, 6·4 %				16·9 %	15·1 %, 18·7 %			
185–400 % FPL	32·6 %	13·8 %	13·0 %, 14·6 %	3·0 %	1·2 %, 4·8 %	0·002*	4·9 %	4·5 %, 5·4 %	−0·4 %	–1·5 %, 0·6 %	0·419	18·6 %	17·7 %, 19·5 %	1·7 %	–0·3 %, 3·7 %	0·102
Less than 185 % FPL	35·7 %	15·0 %	13·9 %, 16·1 %	4·2 %	2·2 %, 6·3 %	< 0·001*	4·5 %	4·0 %, 5·0 %	−0·9 %	–2·1 %, 0·3 %	0·118	19·5 %	18·3 %, 20·7 %	2·6 %	0·3 %, 4·8 %	0·030
Female head of household education																
College degree or more (ref)	30·5 %	12·9 %	12·1 %, 13·6 %				5·2 %	4·8 %, 5·7 %				18·2 %	17·4 %, 19·0 %			
Some college	35·4 %	14·5 %	13·4 %, 15·7 %	1·7 %	0·3 %, 3·1 %	0·018*	4·7 %	4·1 %, 5·3 %	−0·5 %	–1·3 %, 0·2 %	0·158	18·9 %	17·7 %, 20·1 %	0·7 %		0·342
															–0·8 %, 2·2 %	
High school degree or less	39·4 %	15·4 %	13·8 %, 17·1 %	2·6 %	0·8 %, 4·4 %	0·004*	3·8 %	3·2 %, 4·5 %	−1·4 %	–2·2 %, –0·6 %	0·002*	19·8 %	18·1 %, 21·5 %	1·6 %	–0·4 %, 3·5 %	0·103
Household characteristic	No high fructose corn syrup		95 % CI		Difference		95 % CI		P value	Fruit or fruit flavour		95 % CI	Difference		95 % CI	P value
Head of household race/ethnicity																
NH White (ref)	4·8 %		4·4 %, 5·2 %							2·7 %		2·5 %, 3·0 %				
NH Black	4·4 %		3·6 %, 5·3 %		−0·3 %		–1·2 %, 0·6 %		0·479	3·7 %		3·0 %, 4·4 %	0·9 %		0·2 %, 1·7 %	0·009*
NH Asian	5·1 %		3·6 %, 6·6 %		0·3 %		–1·2 %, 1·8 %		0·680	4·2 %		2·8 %, 5·6 %	1·4 %		0·0 %, 2·8 %	0·017
NH other race	4·0 %		2·5 %, 5·6 %		−0·8 %		–2·4 %, 0·9 %		0·394	3·2 %		2·2 %, 4·2 %	0·4 %		–0·6 %, 1·4 %	0·375
Hispanic	4·8 %		4·0 %, 5·7 %		0·0 %		–0·9 %, 1·0 %		0·944	2·7 %		2·1 %, 3·3 %	−0·0 %		–0·7 %, 0·6 %	0·956
Household income																
Greater than 400 % FPL (ref)	5·0 %		4·0 %, 6·0 %							2·5 %		1·9 %, 3·1 %				
185–400 % FPL	4·9 %		4·5 %, 5·4 %		−0·1 %		–1·1 %, 1·0 %		0·915	2·9 %		2·6 %, 3·2 %	0·4 %			0·219
Less than 185 % FPL	4·3 %		3·8 %, 4·8 %		−0·7 %		–1·9 %, 0·4 %		0·184	3·1 %		2·6 %, 3·5 %	0·6 %			0·166
Female head of household education																
College degree or more (ref)	4·9 %		4·5 %, 5·3 %							2·9 %		2·6 %, 3·2 %				
Some college	4·6 %		4·1 %, 5·2 %		−0·3 %		–1·0 %, 0·4 %		0·453	3·0 %		2·6 %, 3·5 %	0·1 %		–0·4 %, 0·7 %	0·596
High school degree or less	4·2 %		3·4 %, 4·9 %		−0·7 %		–1·6 %, 0·1 %		0·113	2·7 %		2·2 %, 3·2 %	−0·2 %		–0·8 %, 0·4 %	0·509

NH, non-Hispanic; FPL, Federal Poverty Level.

Nielsen disclaimer: Authors’ calculations based in part on data reported by Nielsen through its Homescan Services for all food categories, including beverages and alcohol for the 2017 periods across the US market. The Nielsen Company, 2017. Nielsen is not responsible for and had no role in preparing the results reported herein.

* Statistically significantly different after using the Holm method to control the familywise error rate at level 0·05 within each outcome and household characteristic. CI not adjusted for multiple comparisons.

† Model was adjusted for head of household race/ethnicity, female head of household education, federal poverty level index, female head of household age, number of adults in the household, number of children in the household and month of observation. se’s are adjusted for clustering at the household level.

## Discussion

In this study, we found 33·5 % of households with young children in the USA purchased fruit drinks, and they purchased about ¼ cup per d per person. Considering national dietary guidance is to avoid added sugars in early childhood, this is cause for concern. We also found clear disparities in monthly purchases of any fruit drink, regardless of claim type, by sociodemographic characteristics that closely align with the associations seen in studies of young children’s beverage consumption. Studies using data from the National Health and Nutrition Examination Survey (NHANES) have similarly found that children in lower-income, lower-educated, NH Black or Hispanic households are more likely to consume SSB and fruit drinks than their higher-income, higher-educated or NH White counterparts^(1,6,7)^. Given that NH Black and Hispanic households were more likely to purchase any fruit drinks and purchased a larger volume per person per d, we believe these observed purchasing disparities are concerning, particularly considering that SSB consumption in childhood is associated with higher risk of diet-related chronic diseases^(4)^.

In addition to sociodemographic differences in the purchase of any fruit drinks, we also observed some differences among fruit drinks with specific claim types. We found in the weighted models that lower- and middle-income and lower- and middle-education households were more likely to purchase fruit drinks with ‘100 % Vitamin C’ claims. NH Black households were more likely to purchase fruit drinks with fruit or fruit flavour claims and ‘Natural’ claims than NH White households. NH other race and higher-educated households were more likely to purchase fruit drinks with ‘Natural’ claims than NH White and lower-educated households. Some of these differences, particularly for the claims that are less prevalent on fruit drink packaging, are small in absolute terms (e.g. 4·5 % NH white *v*. 6·8 % NH Black households purchasing fruit drinks with a ‘Natural’ claim). However, the types of health claims examined in this study are pervasive in the food supply, so the cumulative effect of differences in exposure to and purchases of products with these claims could be of public health significance. Finally, these findings shed light on patterns across US households with young children, but our study design precludes us from making any causal interpretations about the role of claims in the purchasing decision of the household. We used IP weighting to get closer to isolating the potential impact of claims; however, it was not possible to disentangle claims and other product attributes that may be driving the observed associations. We need more experimental studies to isolate the impact of claims on purchases and qualitative work to better understand the use of specific types of claims in purchasing decisions and how that may differ by sociodemographic characteristics and product categories.

Prior research has clearly demonstrated that FOP nutrition claims on food and beverages increase shoppers’ perceived healthfulness of products and purchase intentions, regardless of the nutritional quality of the product^(33,34)^. Some studies have examined sociodemographic differences in self-reported use of nutrition labels and claims. Still, it appears that the evidence is mixed, and the observed differences are likely dependent on the labelling scheme (e.g. Guiding Stars, stoplight) or claim type^(11,12,35,36)^. One observational study examining differences in food purchases with low or no content claims (e.g. low-fat, no added sugar) by race/ethnicity and socio-economic status found few differences by race/ethnicity but found that middle- and high-socio-economic status households were more likely to purchase products with claims, primarily driven by purchases with low-fat claims^(37)^. On the other hand, we found that households with lower levels of education and income were more likely to purchase fruit drinks with ‘100 % Vitamin C’ claims. Prior studies have found that individuals with lower levels of education and income may be less likely to use the nutrition facts panel and ingredients list^(11)^, so it is possible these groups may be more likely to mistake the ‘100 % Vitamin C’ claim on a fruit drink for a ‘100 % juice’ claim and not check the ingredients list to confirm that a product is, in fact, 100 % juice and not a sugary drink. Another observational study examining differences in purchases of fruit drinks and 100 % juice with certain claims found no significant differences by household income, Supplemental Nutrition Assistance Program participation, or Special Supplemental Nutrition Program for Women, Infants, and Children participation but found that Hispanic households purchased more fruit drinks with natural claims and NH Black households purchased more 100 % juice with sugar, vitamin C, and implied natural claims^(10)^. This is consistent with our findings that NH Black households were more likely to purchase fruit drinks with fruit or fruit flavour and ‘Natural’ claims. Some research suggests rapid growth in NH Black household purchases of organic products in recent years^(38)^, perhaps representing an interest in products with claims related to products being natural, pure or organic, but we cannot determine the mechanism for these observed associations from this study. It is also possible that these claims are not driving differences in purchasing observed but, instead, certain claims may be more likely to be present on specific brands of fruit drinks that are target-marketed to communities of colour^(39)^. However, given the observational study design, we are unable to disentangle the effects of individual claims from other product attributes such as brands or price or taste preferences.

In addition to observational studies, some experimental studies have examined the differential impact of FOP claims by shopper sociodemographic characteristics. One study examining the impact of three different claim types on parents’ selection of a fruit drink for their child found no significant differences in the impact of claims by parent income, race/ethnicity or education^(17)^. Among all parents in this study, claims led parents to select less healthy drinks for their children and caused misperceptions about the nutritional quality of fruit drinks^(17)^. Another experimental study examined the impact of qualified health claims on green tea purchase intentions and found that Black and Hispanic participants reported greater purchasing intentions than White participants^(39)^. The current study adds to existing research by examining real-life purchases of products with specific claims among different sociodemographic groups and by using IP weighting to account for the selectivity of being a fruit drink purchaser.

Taken together, the existing experimental and observational research suggests that claims on fruit drinks lead parents to make less healthy choices for their children^(17,18)^, claims are prevalent on almost all fruit drinks purchased by households with young children^(8,10)^ and sociodemographic groups at greatest risk of diet-related chronic disease are more likely to purchase fruit drinks than other groups. The research on which sociodemographic groups may be more likely to purchase fruit drinks with specific claims is mixed and does not account for other product-level attributes that may have influenced the purchase. Despite these limitations, there are still solutions that could reduce observed disparities in fruit drink purchases and consumption in early childhood such as prominent FOP disclosures of sweeteners on any product with a nutrition claim, prominent FOP percent juice disclosures on fruit drinks and FOP health warnings on sugary drinks^(40,41)^. Some of these strategies such as FOP sugar-related disclosures have been demonstrated to reduce the appeal of SSB^(9,42)^, even when claims are present on the FOP^(9)^. However, other strategies will be needed to reduce disparities in fruit drink and SSB consumption such as policies that address pricing, availability and targeted marketing of SSB including fruit drinks^(43)^.

This study has several strengths including the use of a unique dataset of real-world purchasing data from a large sample of households with young children linked to FOP nutrition claims data to examine characteristics associated with purchases of fruit drinks with specific FOP claims. Also, our findings align with patterns identified between sociodemographic characteristics and fruit drink or SSB consumption using NHANES and the Feeding Infants and Toddler Study^(1,6,44)^. This study also moves the body of research surrounding claims and purchases forward by using IP weights to account for selection bias of being a purchaser of fruit drinks. There are some important limitations to this work. First, our study design precludes us from making any causal claims about the impact of FOP claims on household purchasing decisions and from accounting for other product-level characteristics that may have influenced purchasing decisions such as brand or price. Also, we were not able to use Nielsen’s survey weights to create a nationally representative sample because the weights cannot be applied while simultaneously accounting for clustering of observations at the household level. Additionally, we used purchasing data from 2017 because these were the most recent data available when products were coded for claims; however, compared with a similar study using data from 2012 to 2013^(10)^, the most common claims were consistent across our dataset and theirs, indicating the claim profiles on fruit drinks are likely not changing drastically over time. We also cannot definitively determine the health effects of the disparities in purchases observed. Finally, our sample is limited to households in the USA. While some research suggests that differences in nutrition claims or label use by sociodemographic characteristics such as income are relatively consistent across country contexts^(35)^, we cannot determine if the associations we observed would apply to settings outside of the USA.

## Conclusions

There are stark sociodemographic disparities in purchases of fruit drinks among households with young children in the USA, with the sociodemographic groups often at greatest risk of diet-related chronic diseases being most likely to purchase fruit drinks. There are some disparities in purchases of fruit drinks with specific FOP nutrition claims, particularly of fruit drinks with ‘100 % Vitamin C’ and ‘Natural’ claims, suggesting these may be important targets for regulatory efforts. However, experimental evidence is needed to understand the impact of potential policy and regulatory solutions, such as restricting certain types of claims on products with high levels of added sugar, on parents’ fruit drink purchases and perceptions. Reforming regulations around FOP claims in the USA could be one of many policy strategies needed to reduce disparities in fruit drink intake and diet-related chronic diseases among children.

## Supplementary material

For supplementary material accompanying this paper visit https://doi.org/10.1017/S1368980023000691. click here to view supplementary material

## References

[1] Mendez MA , Miles DR , Poti JM et al. (2019) Persistent disparities over time in the distribution of sugar-sweetened beverage intake among children in the United States. Am J Clin Nutr 109, 79–89. 30535176 10.1093/ajcn/nqy123 PMC6698637

[2] Kay MC , Welker EB , Jacquier EF et al. (2018) Beverage consumption patterns among infants, young children (0–47·9 Months): data from the feeding infants, toddlers study, 2016. Nutrients 10, 825. 29949886 10.3390/nu10070825 PMC6073729

[3] Herrick KA , Fryar CD , Hamner HC et al. (2020) Added sugars intake among US infants and toddlers. J Acad Nutr Diet 120, 23–23. 31735600 10.1016/j.jand.2019.09.007 PMC7512119

[4] Bleich SN & Vercammen KA (2018) The negative impact of sugar-sweetened beverages on children’s health: an update of the literature. BMC Obes 5, 6. 29484192 10.1186/s40608-017-0178-9 PMC5819237

[5] Harris JL , Frazier W , Kumanyika S et al. (2019) Increasing Disparities in Unhealthy Food Advertising Targeted to Hispanic and Black Youth. Storrs, CT: UConn Rudd Center for Food Policy & Obesity.

[6] Demmer E , Cifelli CJ , Houchins JA et al. (2018) Ethnic disparities of beverage consumption in infants and children 0–5 years of age; National Health and Nutrition Examination Survey 2011 to 2014. Nutr J 17, 78. 30134909 10.1186/s12937-018-0388-0 PMC6106834

[7] Grimes CA , Szymlek-Gay EA & Nicklas TA (2017) Beverage consumption among U.S. children aged 0–24 months: National Health and Nutrition Examination Survey (NHANES). Nutrients 9, 264. 28335374 10.3390/nu9030264 PMC5372927

[8] Duffy EW , Hall MG , Dillman Carpentier FR et al. (2021) Nutrition claims on fruit drinks are inconsistent indicators of nutritional profile: a content analysis of fruit drinks purchased by households with young children. J Acad Nutr Diet 121, 36–46.e4. 32978105 10.1016/j.jand.2020.08.009 PMC7752796

[9] Hall MG , Lazard AJ , Grummon AH et al. (2020) The impact of front-of-package claims, fruit images, and health warnings on consumers’ perceptions of sugar-sweetened fruit drinks: three randomized experiments. Prev Med 132, 105998. 31982477 10.1016/j.ypmed.2020.105998 PMC7085890

[10] Musicus AA , Hua SV , Moran AJ et al. (2022) Front-of-package claims & imagery on fruit-flavored drinks and exposure by household demographics. Appetite 171, 105902. doi: 10.1016/j.appet.2021.105902. 34968559 PMC8821268

[11] Bleich SN & Wolfson JA (2015) Differences in consumer use of food labels by weight loss strategies and demographic characteristics. BMC Public Health 15, 1275. 26690655 10.1186/s12889-015-2651-z PMC4687126

[12] Christoph MJ , Larson N , Laska MN et al. (2018) Nutrition facts panels: who uses them, what do they use, and how does use relate to dietary intake? J Acad Nutr Diet 118, 217–228. 29389508 10.1016/j.jand.2017.10.014 PMC5797995

[13] Calderon J , Ayala GX , Elder JP et al. (2017) What happens when parents and children go grocery shopping? An observational study of Latino Dyads in Southern California, USA. Health Educ Behav 44, 5–12. 27162238 10.1177/1090198116637602 PMC5435120

[14] Castro IA , Calderon J & Ayala GX (2017) Who is influencing whom? Latino Parent-Child request interactions and product purchases in food retail environments. Soc Mar Q 23, 155–168. 29081718 10.1177/1524500416686038 PMC5659367

[15] Abrams KM , Evans C & Duff BR (2015) Ignorance is bliss. How parents of preschool children make sense of front-of-package visuals and claims on food. Appetite 87, 20–29. 25510529 10.1016/j.appet.2014.12.100

[16] Munsell CR , Harris JL , Sarda V et al. (2016) Parents’ beliefs about the healthfulness of sugary drink options: opportunities to address misperceptions. Public Health Nutr 19, 46–54. 25757372 10.1017/S1368980015000397 PMC10271054

[17] Hall MG , Lazard AJ , Higgins ICA et al. (2022) Nutrition-related claims lead parents to choose less healthy drinks for young children: a randomized trial in a virtual convenience store. Am J Clin Nutr 115, 1144–1154. 35040866 10.1093/ajcn/nqac008 PMC8971006

[18] Dixon H , Scully M , Niven P et al. (2014) Effects of nutrient content claims, sports celebrity endorsements and premium offers on pre-adolescent children’s food preferences: experimental research. Pediatr Obes 9, e47–57. 23630014 10.1111/j.2047-6310.2013.00169.x

[19] US Food and Drug Administration (2023) FDA Nutrition Innovation Strategy. https://www.fda.gov/food/food-labeling-nutrition/fda-nutrition-innovation-strategy (accessed October 2019).

[20] Ng SW & Popkin BM (2014) The Healthy Weight Commitment Foundation pledge: calories purchased by U.S. households with children, 2000–2012. Am J Prev Med 47, 520–30. 25240968 10.1016/j.amepre.2014.05.030 PMC4171651

[21] Goodman MJ (2017) The ‘Natural’ *v.* ‘Natural Flavors’ conflict in food labeling: a regulatory viewpoint. Food Drug Law J 72, 78–102. 29140655

[22] Center for Science in the Public Interest (2018) Re: FDA-2018-N-238; The Food and Drug Administration’s Comprehensive, Multi-Year Nutrition Innovation Strategy; Public Meeting; Request for Comments. https://cspinet.org/sites/default/files/attachment/nis-cspi-comments-withappendix_0.pdf (accessed February 2021).

[23] André Q , Chandon P & Haws K (2019) Healthy through presence or absence, nature or science? A framework for understanding front-of-package food claims. J Public Policy Market 38, 172–91.

[24] Zick CD , Stevens RB & Bryant WK (2011) Time use choices and healthy body weight: a multivariate analysis of data from the American Time Use Survey. Int J Behav Nutr Phys Act 8, 84. 21810246 10.1186/1479-5868-8-84 PMC3199736

[25] Ollberding NJ , Wolf RL & Contento I (2011) Food label use and its relation to dietary intake among US adults. J Am Diet Assoc 111, S47–S51. 21515135 10.1016/j.jada.2011.03.009

[26] Roth WD (2016) The multiple dimensions of race. Ethn Racial Stud 39, 1310–38.

[27] Smith N , Martinez RA , Andrabi N et al. (2020) Beyond the Boxes, Part 2: Defining Race. https://iaphs.org/beyond-the-boxes-part-2-defining-race-and-ethnicity/ (accessed February 2021).

[28] StataCorp (2019) Stata Statistical Software: Release 16. College Station, TX: StataCorp LLC.

[29] Hogan JW & Lancaster T (2004) Instrumental variables and inverse probability weighting for causal inference from longitudinal observational studies. Stat Methods Med Res 13, 17–48. 14746439 10.1191/0962280204sm351ra

[30] Seaman SR & White IR (2013) Review of inverse probability weighting for dealing with missing data. Stat Methods Med Res 22, 278–295. 21220355 10.1177/0962280210395740

[31] Cole SR & Hernán MA (2008) Constructing inverse probability weights for marginal structural models. Am J Epidemiol 168, 656–64. 18682488 10.1093/aje/kwn164 PMC2732954

[32] Chen S-Y , Feng Z & Yi X (2017) A general introduction to adjustment for multiple comparisons. J Thorac Dis 9, 1725. 28740688 10.21037/jtd.2017.05.34 PMC5506159

[33] Oostenbach LH , Slits E , Robinson E et al. (2019) Systematic review of the impact of nutrition claims related to fat, sugar and energy content on food choices and energy intake. BMC Public Health 19, 1296. 31615458 10.1186/s12889-019-7622-3 PMC6794740

[34] Kaur A , Scarborough P & Rayner M (2017) A systematic review, and meta-analyses, of the impact of health-related claims on dietary choices. Int J Behav Nutr Phys Act 14, 93. 28697787 10.1186/s12966-017-0548-1 PMC5505045

[35] Campos S , Doxey J & Hammond D (2011) Nutrition labels on pre-packaged foods: a systematic review. Public Health Nutr 14, 1496–506. 21241532 10.1017/S1368980010003290

[36] Graham DJ , Heidrick C & Hodgin K (2015) Nutrition label viewing during a food-selection task: front-of-package labels *v.* nutrition facts labels. J Acad Nutr Diet 115, 1636–1646. 25887785 10.1016/j.jand.2015.02.019

[37] Taillie LS , Ng SW , Xue Y et al. (2017) No fat, no sugar, no salt… No problem? Prevalence of ‘low-content’ nutrient claims and their associations with the nutritional profile of food and beverage purchases in the United States. J Acad Nutr Diet 117, 1366–1374. e6. 28330730 10.1016/j.jand.2017.01.011 PMC5573644

[38] Berhaupt-Glickstein A , Hooker NH & Hallman WK (2019) Qualified health claim language affects purchase intentions for green tea products in the United States. Nutrients 11, 921. 31022930 10.3390/nu11040921 PMC6521090

[39] Pomeranz JL & Harris JL (2020) Children’s fruit ‘Juice’ drinks and FDA regulations: opportunities to increase transparency and support public health. Am J Public Health 110, 871–80. 32298182 10.2105/AJPH.2020.305621 PMC7204473

[40] Harris J , Kibwana-Jaff A & Phaneuf L (2020) Sugary Drink FACTS 2020 Sugary Drink Advertising to Youth: Continued Barrier to Public Health Progress. UConn Rudd Center for Food Policy & Obesity. https://www.sugarydrinkfacts.org/resources/Sugary%20Drink%20FACTS%202020/Sugary_Drink_FACTS_Full%20Report_final.pdf (accessed July 2020).

[41] Roberto CA , Wong D , Musicus A et al. (2016) The influence of sugar-sweetened beverage health warning labels on parents’ choices. Pediatrics 137, e20153185. 26768346 10.1542/peds.2015-3185

[42] Krieger J , Bleich SN , Scarmo S et al. (2020) Sugar-sweetened beverage reduction policies: progress and promise. Annu Rev Public Health 42, 439–461. 10.1146/annurev-publhealth-090419-103005 33256536

[43] Welker EB , Jacquier EF , Catellier DJ et al. (2018) Room for improvement remains in food consumption patterns of young children aged 2–4 years. J Nutr 148, 1536S–1546S. 29878237 10.1093/jn/nxx053 PMC6126636

