# Supplementary material for: Sociodemographic disparities in purchases of fruit drinks with policy relevant front-of-package nutrition claims
Source: Public Health Nutr. 2023 May 22;26(8):1585–95. doi: 10.1017/S1368980023000691 (PMC10410375; doi:10.1017/S1368980023000691)
Supplement: Supplementary file 1 [file S1368980023000691sup.zip › S1368980023000691sup001.docx]

**S1 File: Inverse Probability Weights Documentation**

We used logistic regression to predict the probability of purchasing any fruit drinks for each household-month observation using household-level covariates that we expected to be predictors of fruit drink purchases based on existing evidence of demographic characteristics associated with sugar-sweetened beverage and fruit drink consumption in early childhood(1, 2, 6, 7).

P (purchase any fruit drinks) = head of household race/ethnicity + household FPL index + number of children in the household + number of adults in the household + female head household education + female head of household age + month

Head of household race/ethnicity: indicator variable with non-Hispanic (NH) White households as referent

FPL index: 3-level categorical variable with greater than 400% FPL as referent

Number of children: indicator variable for number of children (less than 18 y) in household

Number of adults: indicator variable for number of adults in household

Female head of household education: indicator variable with college education or more as referent

Female head of household age: indicator variable using quartiles of age with less than 34 years as referent

Month: indicator variable for month

We then confirmed that no fruit drink purchasing households had a predicted probability of purchase of 0 based on this model’s output. We also stabilized the weights to increase precision by dividing the marginal probability of either purchasing or not purchasing any fruit drinks in a given month by the predicted probabilities from the logistic regression model for fruit drink purchasing or non-purchasing households, respectively (Supplemental Table 1) (30, 45).

Weights were constructed for each household month observation by taking the inverse of the predicted probability of purchasing any fruit drinks from the logistic regression model. Truncation of weights was not warranted as there were no extreme values. We also compared the distribution of covariates of interest in the weighted and unweighted sample to assess whether the first step logistic regression model was adequately specified(45). After weighting, all covariates are balances across fruit drink purchasing and fruit drink non-purchasing households (Supplemental Table 2).
