# Supplementary material for: Sociodemographic disparities in purchases of fruit drinks with policy relevant front-of-package nutrition claims
Source: Public Health Nutr. 2023 May 22;26(8):1585–95. doi: 10.1017/S1368980023000691 (PMC10410375; doi:10.1017/S1368980023000691)
Supplement: Supplementary file 1 [file S1368980023000691sup.zip › S1368980023000691sup002.docx]

**S1 Table** Descriptive statistics of inverse probability weights and stabilized inverse probability weights

|  |  | **Mean** | **SD** | **Min** | **Max** |
| --- | --- | --- | --- | --- | --- |
| **Fruit drink purchasing households** | IPW | 3.0 | 0.8 | 1.4 | 7.4 |
|  | Stabilized IPW | 1.0 | 0.3 | 0.5 | 2.2 |
| **Fruit drink non-purchasing households** | IPW | 3.3 | 0.9 | 1.4 | 7.4 |
|  | Stabilized IPW | 1.0 | 0.1 | 0.75 | 2.1 |
