# Supplementary material for: Sociodemographic disparities in purchases of fruit drinks with policy relevant front-of-package nutrition claims
Source: Public Health Nutr. 2023 May 22;26(8):1585–95. doi: 10.1017/S1368980023000691 (PMC10410375; doi:10.1017/S1368980023000691)
Supplement: Supplementary file 1 [file S1368980023000691sup.zip › S1368980023000691sup003.docx]

**S2 Table:** Distribution of covariates in unweighted and weighted sample of household month observations

| **Characteristic** | | **Unweighted Sample** | | **Weighted Sample** | |
| --- | --- | --- | --- | --- | --- |
|  |  | **Fruit drink purchasing households** | **Fruit drink non-purchasing households** | **Fruit drink purchasing households** | **Fruit drink non-purchasing households** |
| **Head of household race/ethnicity** | |  |  |  |  |
|  | NH White | 65.7% | 72.7% | 70.3% | 70.3% |
|  | NH Black | 14.5% | 6.8% | 9.4% | 9.4% |
|  | NH Asian | 3.6% | 6.0% | 5.2% | 5.2% |
|  | NH other race | 3.0% | 2.8% | 2.9% | 2.9% |
|  | Hispanic | 13.3% | 11.7% | 12.2% | 12.2% |
| **Female head of household education** | |  |  |  |  |
|  | College or more | 54.5% | 63.2% | 60.2% | 60.2% |
|  | Some college | 29.3% | 25.0% | 26.5% | 26.5% |
|  | High school graduate or less | 16.2% | 11.8% | 13.3% | 13.3% |
| **Household income** | |  |  |  |  |
|  | >400% FPL | 11.6% | 16.8% | 15.0% | 15.0% |
|  | 185%-400% FPL | 54.3% | 56.7% | 56.0% | 55.9% |
|  | <185% FPL | 34.1% | 26.5% | 29.1% | 29.0% |
| **Female head of household age** | |  |  |  |  |
|  | Less than 34 years | 30.9% | 31.9% | 31.3% | 31.5% |
|  | 34-36 years | 21.9% | 22.7% | 22.6% | 22.4% |
|  | 37-40 years | 21.6% | 22.4% | 22.0% | 22.1% |
|  | Greater than 40 years | 25.7% | 23.1% | 24.1% | 24.0% |
| **Number of adults in household** | |  |  |  |  |
|  | **1** | 4.1% | 3.4% | 3.6% | 3.6% |
|  | **2** | 77.8% | 81.4% | 80.1% | 80.2% |
|  | **3** | 11.2% | 8.8% | 9.7% | 9.7% |
|  | **4** | 5.2% | 4.7% | 4.9% | 4.9% |
|  | **5** | 1.2% | 1.2% | 1.2% | 1.2% |
|  | **6-8** | 0.5% | 0.4% | 0.5% | 0.4% |
| **Number of children in household** | |  |  |  |  |
|  | **1** | 27.0% | 34.1% | 31.7% | 31.7% |
|  | **2** | 40.0% | 38.9% | 39.2% | 39.3% |
|  | **3** | 21.3% | 17.3% | 18.7% | 18.7% |
|  | **4** | 8.5% | 7.0% | 7.5% | 7.5% |
|  | **5-7** | 3.3% | 2.7% | 2.9% | 2.9% |
| **Month** | |  |  |  |  |
|  | **1** | 7.3% | 8.2% | 7.2% | 8.1% |
|  | **2** | 7.4% | 8.4% | 7.4% | 8.4% |
|  | **3** | 7.7% | 8.7% | 7.7% | 8.7% |
|  | **4** | 9.5% | 8.0% | 9.4% | 8.0% |
|  | **5** | 8.9% | 8.3% | 8.9% | 8.3% |
|  | **6** | 9.2% | 8.0% | 9.2% | 8.0% |
|  | **7** | 10.3% | 7.6% | 10.3% | 7.6% |
|  | **8** | 8.5% | 8.4% | 8.5% | 8.4% |
|  | **9** | 8.8% | 8.3% | 8.9% | 8.3% |
|  | **10** | 7.4% | 8.8% | 7.4% | 8.8% |
|  | **11** | 7.4% | 8.8% | 7.4% | 8.8% |
|  | **12** | 7.7% | 8.5% | 7.8% | 8.5% |
